# Boosted Tetracycline and Cr(VI) Simultaneous Cleanup over Z-Scheme WO_3_/CoO p-n Heterojunction with 0D/3D Structure under Visible Light

**DOI:** 10.3390/molecules28124727

**Published:** 2023-06-13

**Authors:** Changyu Lu, Delu Cao, Hefan Zhang, Luning Gao, Weilong Shi, Feng Guo, Yahong Zhou, Jiahao Liu

**Affiliations:** 1 Hebei Province Collaborative Innovation Center for Sustainable Utilization of Water Resources and Optimization of Industrial Structure, Hebei Province Key Laboratory of Sustained Utilization & Development of Water Recourse, School of Water Resource and Environment, Hebei Geo University, Shijiazhuang 050031, China; pzpzlxl@163.com (C.L.); caodelu7@163.com (D.C.); zhang020518@163.com (H.Z.); gaoluning1997@163.com (L.G.); gfeng0105@126.com (F.G.); 2 School of Material Science and Engineering, Jiangsu University of Science and Technology, Zhenjiang 212003, China; shiwl@just.edu.cn

**Keywords:** photocatalysis, Z-scheme, WO_3_/CoO, 0D/3D, p-n heterojunction, tetracycline and Cr(VI)

## Abstract

In this study, a Z-Scheme WO_3_/CoO p-n heterojunction with a 0D/3D structure was designed and prepared via a simple solvothermal approach to remove the combined pollution of tetracycline and heavy metal Cr(VI) in water. The 0D WO_3_ nanoparticles adhered to the surface of the 3D octahedral CoO to facilitate the construction of Z-scheme p-n heterojunctions, which could avoid the deactivation of the monomeric material due to agglomeration, extend the optical response range, and separate the photogenerated electronhole pairs. The degradation efficiency of mixed pollutants after a 70 min reaction was significantly higher than that of monomeric TC and Cr(VI). Among them, a 70% WO_3_/CoO heterojunction had the best photocatalytic degradation effect on the mixture of TC and Cr(VI) pollutants, and the removing rate was 95.35% and 70.2%, respectively. Meanwhile, after five cycles, the removal rate of the mixed pollutants by the 70% WO_3_/CoO remained almost unchanged, indicating that the Z-scheme WO_3_/CoO p-n heterojunction has good stability. In addition, for an active component capture experiment, ESR and LC-MS were employed to reveal the possible Z-scheme pathway under the built-in electric field of the p-n heterojunction and photocatalytic removing mechanism of TC and Cr(VI). These results offer a promising idea for the treatment of the combined pollution of antibiotics and heavy metals by a Z-scheme WO_3_/CoO p-n heterojunction photocatalyst, and have broad application prospects: boosted tetracycline and Cr(VI) simultaneous cleanup over a Z-scheme WO_3_/CoO p-n heterojunction with a 0D/3D structure under visible light.

## 1. Introduction

Since the 1940s, tetracycline (TC) has been recognized as an excellent antibiotic and has been widely used in human therapy, veterinary medicine, agriculture, and other fields [1,2,3]. However, the abuse of TC leads to its accumulation in the water environment [4]. Due to the fact that it is highly toxic, resistant, and difficult to biodegrade, TC accumulates in water for long periods, creating potential environmental stress [5,6,7]. In addition, heavy metals often coexist with antibiotic contaminants in water environments and cause irreversible damage to humans and other organisms [8,9,10]. The most typical is Cr (VI) contamination in water, which can be carcinogenic and cause lasting harm to the environment when ingested by humans [11,12]. Unfortunately, the combined toxicity of antibiotics and heavy metals is much higher than that of a single contaminant [13,14,15]. Antibiotic molecules contain a large number of carboxyl, hydroxyl, amino, heterocyclic, and other groups or electron donors, which can be complexed with heavy metal ions to change the environmental behavior and toxicological effects of pollutants in the complex pollution system, and may produce synergistic and antagonistic complex toxicological effects on micro-organisms, animals, and plants [16]. Therefore, seeking a new efficient, straightforward technology free of secondary pollution is essential for treating TC and Cr (VI) residues in water [17,18,19]. Semiconductor photocatalysis is an advanced redox technology that uses clean solar power as a power source to treat organic pollutants and heavy metal pollution in water [20,21,22]. Photocatalysis offers many advantages over conventional methods, including a simple preparation process, wide application range, good catalytic effect, and no secondary pollution [23,24], and it is considered by many researchers to be a superior and environmentally friendly purification technology [25,26,27,28].

Since Fujishu and Honda reported in 1972 that TiO_2_ has an excellent photocatalytic effect under ultraviolet light irradiation, traditional photocatalysis such as TiO_2_ and ZnO has been extensively studied [29]. However, TiO_2_ and ZnO have a wide band gap, extremely low visible light utilization, and fast electron–hole recombination, severely limiting their photocatalytic activity and effective utilization of visible light [30]. The p-type semiconductor cobalt oxide (CoO) is an excellent photocatalytic material with a smaller band gap of 2.2–2.6 eV, efficiently utilizing visible light [28,31]. However, the photocatalytic activity of pure CoO was limited by the high recombination rate of separate electron–hole pairs and inactivation due to agglomeration [32]. Fortunately, the construction of heterojunction nanocomposites can effectively solve the above problems and improve their photocatalytic efficiency [33,34]. In addition, three-dimensional octahedral CoO has the advantages of good stability, easy preparation, and good stability of its electrochemical reactions, and good prospects for the construction of heterojunctions [35,36,37]. For example, Zou et al. present a novel 3D/3D composite structure in which the 3D CoO acts as an active and stable ion diffusion channel. At the same time, the hollow NiCo LDH provides abundant redox sites. Chen et al. [38] reported the oxygen-vacancy-induced construction of the CoO/h-TiO_2_ Z-scheme heterostructures, which can be used for photocatalytic hydrogen production from water decomposition. Wang et al. [39] designed a novel one-dimensional/two-dimensional (1D/2D) core-shell cobalt monoxide/nickel-cobalt layered double hydroxide (CoO/Nico-LDH) heterojunction, which can avoid agglomeration, promote the transfer of photogenerated carriers, and expand the light absorption range, effectively enhancing the photocatalytic performance. As a conventional photocatalytic material, WO_3_ has a band gap of about 2.5–2.8 eV [40], can absorb visible light at nearly 500 nm, and has good photocatalytic performance in visible light [41,42,43]. Cao et al. [44] reported the electron transfer mechanism of noble-metal-free WO_3_@ZnIn_2_S_4_ S-scheme heterojunction photocatalysts. This provides a new idea for improving the photocatalytic efficiency of WO_3_ through the formation of heterojunctions [45]. Consequently, Z-scheme p-n heterojunctions can be constructed using WO_3_ as 0D nanoparticles attached to 3D octahedral CoO [46,47,48]. This can not only broaden the visible light response range of the monomeric material but also effectively improve the separation efficiency of the photogenerated carriers and the stability of the material [49,50,51].

In this article, Z-scheme WO_3_/CoO p-n heterojunctions were designed and synthesized via a simple solvothermal approach. The XRD, TEM, XPS, and UV-vis measurement were chosen to characterize the microstructure, surface chemistry, and photoelectrochemical properties of WO_3_/CoO p-n heterojunctions. In addition, the reusability and stability of the Z-scheme WO_3_/CoO p-n heterojunctions were tested by five cycles. Finally, the potential photocatalytic mechanisms in the photocatalytic degradation of TC were elaborated.

## 2. Results and Discussion

### 2.1. XRD Analysis

**Figure 1:**
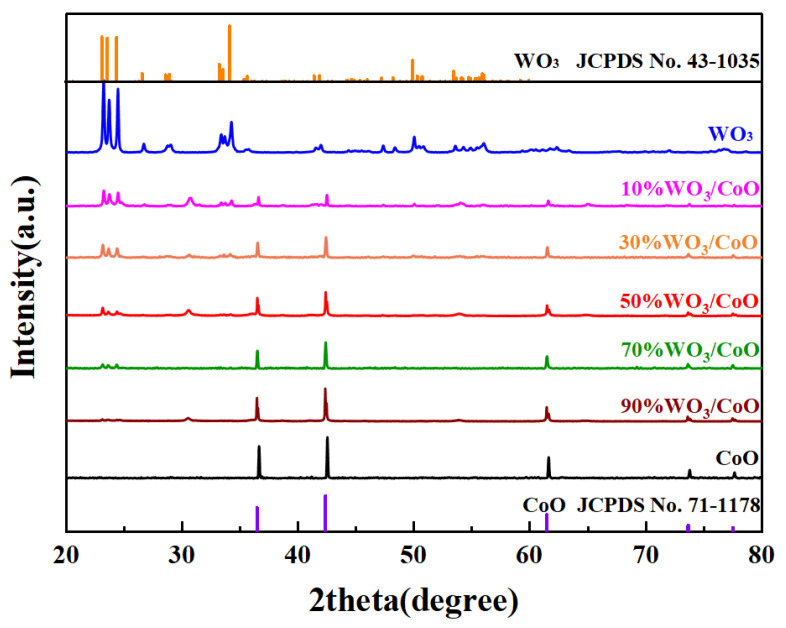
XRD patterns of CoO, WO_3_, and WO_3_/CoO heterojunctions.

X-ray diffraction (XRD) was used to study the phase structure of the as-synthesized WO_3_, CoO, and various WO_3_/CoO heterojunctions. As indicated in Figure 1, the diffraction peaks of pure WO_3_ at 2θ = 23.12°, 23.59°, 24.38°, 33.26°, 33.57°, 34.15°, and 49.95° corresponded to the (002), (020), (200), (022), (−202), (202), and (140) crystal planes of the monoclinic phase (JCPDS card NO. 43-1035), respectively [52,53]. Moreover, the characteristic peaks at 36.48° and 42.37° in pure CoO and different WO_3_/CoO heterojunctions are normalized to the (111) and (200) planes of cubic phase CoO (JCPDS 71-1178) [54,55]. With the construction of the WO_3_/CoO heterojunction, the characteristic peak of WO_3_ increased with the increase of content, and no other obvious peaks were found, indicating that no impurities were observed during the recombination process. It was worth noting that the prominent diffraction peaks of WO_3_ and CoO had slightly shifted to the middle, which can be ascribed to the metal ion bonding in creating heterojunctions between WO_3_ and CoO.

### 2.2. Morphology

**Figure 2:**
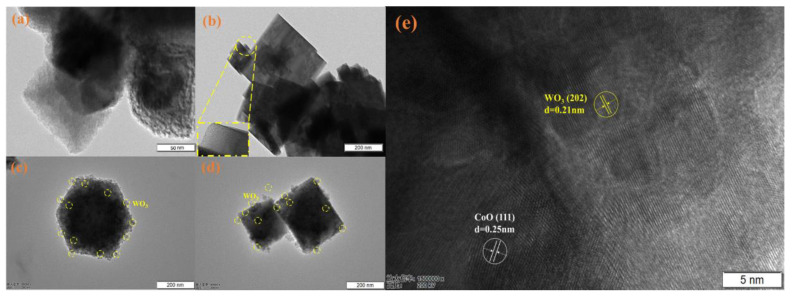
TEM of (**a**) CoO, (**b**) WO_3_; and (**c**–**e**) TEM and HR-TEM of 70% WO_3_/CoO.

SEM was employed to observe the morphology of the WO_3_, CoO, and 70% WO_3_/CoO heterojunction, and shown in Figure S1. As illustrated in Figure S1a, CoO possessed an octahedral form with a smooth surface and an average size of about 200 nm. WO_3_ appeared as a nanosheet structure of agglomeration with a side length of about 350 nm. In Figure S1c, the morphology and particle size of the WO_3_/CoO heterojunction was very similar to that of pure CoO, indicating that the addition of WO_3_ had no significant change in the morphology and particle size of CoO. After the combination of WO_3_ and CoO, WO_3_ nanoparticles adhered to the surface of the octahedral CoO, indicating that WO_3_/CoO heterojunction was well-constructed, whereafter the specific morphology of the WO_3_, CoO, and the 70% WO_3_/CoO heterojunction was further analyzed by TEM. Figure 2a clearly showed that the octahedral CoO was stacked together and the size was in accord with the above SEM results. As shown in Figure 2b, WO_3_ presented the nanosheet structure with accumulation and in agreement with the morphology of SEM. As shown in Figure 2c,d, it can be seen that the CoO composite material still presented a representative octahedral shape, and WO_3_ changed from sheet to granular during preparation and deposited on the surface of the octahedral CoO. Figure 2e showed the HR-TEM images of the 70% WO_3_/CoO heterojunction material for a lattice study. It can be seen from the figure that the 0.25 nm lattice fringe spacing corresponded to the (111) plane of the CoO composite material based on JCPDS 71-1178, while the 0.21 nm lattice spacing corresponded to the (202) plane of the WO_3_ material according to the standard card (JCPDS 43-1035) [52,55].

### 2.3. XPS

**Figure 3:**
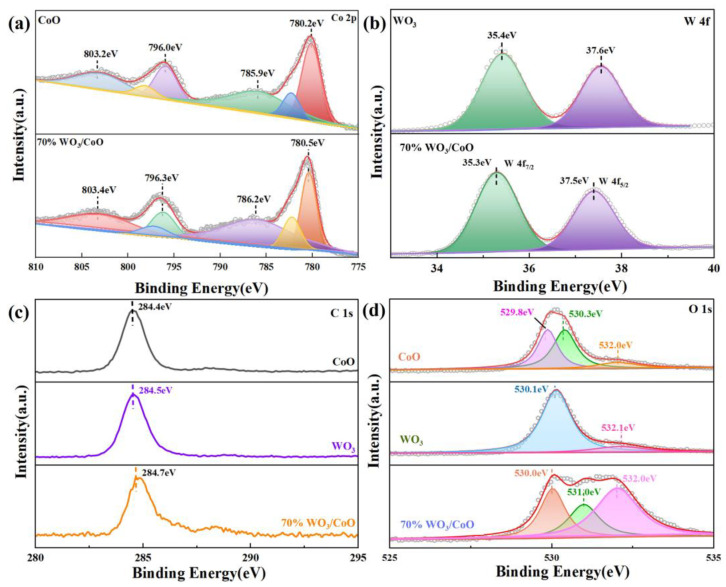
High-resolution spectra: (**a**) Co 2p, (**b**) W 4f, (**c**) C 1s, and (**d**) O 1s of the 70% WO_3_/CoO heterojunction.

The surface chemical states, chemical composition, and molecular structure of the as-prepared photocatalysts were examined by XPS spectroscopy. As shown in Figure S2, the XPS survey spectrum of 70%WO_3_/CoO consists of Co, W, and O, with all characteristic peaks of WO_3_ and CoO, and no impurities other than carbon were found in the spectrum element, consistent with the XRD results. The C1s peak observed at 284.4 eV can be attributed to the signal from carbon during the subsequent processing of the measurement of the scanning spectrum. Figure 3a displays the high-resolution Co 2p spectrum. As shown in the figure, the two main peaks were located at 780.2 and 796.3 eV, while the two satellite peaks were located near 786.2 eV and 802.9 eV, which correspond to Co 2p_1/2_ and Co 2p_3/2_, manifesting the occurrence of Co^2+^ [56]. As depicted in Figure 3b, two peaks of 35.4 eV and 37.4 eV are attributed to W4 f_7/2_ and W4 f_5/2_ in WO_3_, respectively, belonging to the W^6+^ in WO_3_. After the combination of CoO and WO_3_, the W 4f_7/2_ and W 4f_5/2_ of WO_3_/CoO drift to 35.2 eV and 37.0 eV, which were slightly lower than the W 4f_7/2_ and W 4f_5/2_ of pure WO_3_ [57]. On the contrary, the Co 2p peaks of WO_3_/CoO were slightly higher than those of pure CoO. The above results indicated that electrons were transferred from the CB of WO_3_ to the VB of CoO with the following Z-scheme pathway, meaning the Z-scheme WO_3_/CoO heterostructure had been successfully constructed [58,59]. Furthermore, the O 1s spectrum of WO_3_/CoO could be resolved as triple peaks at 530.0 eV, 531.0 eV, and 532.0 eV, as shown in Figure 3d. Among them, the formation of the characteristic peak at 530.0 eV was due to the typical lattice oxygen in CoO and WO_3_ [60,61], while the characteristic peaks were also observed at 531.0 eV and 532.0 eV, possibly due to the combination of O in H_2_O on the surface of WO_3_/CoO with active species (•OH and •O_2_^−^) [62,63].

### 2.4. UV-Vis

**Figure 4:**
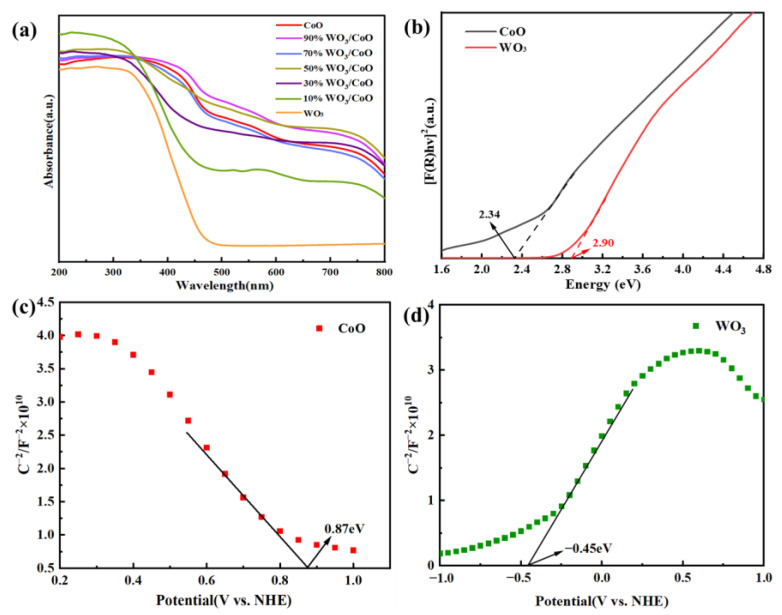
(**a**) UV-Vis DRS of CoO, WO_3_, and WO_3_/CoO; (**b**) Tauc plot; and (**c**,**d**) Mott–Schottky of CoO and WO_3_.

**Figure 5:**
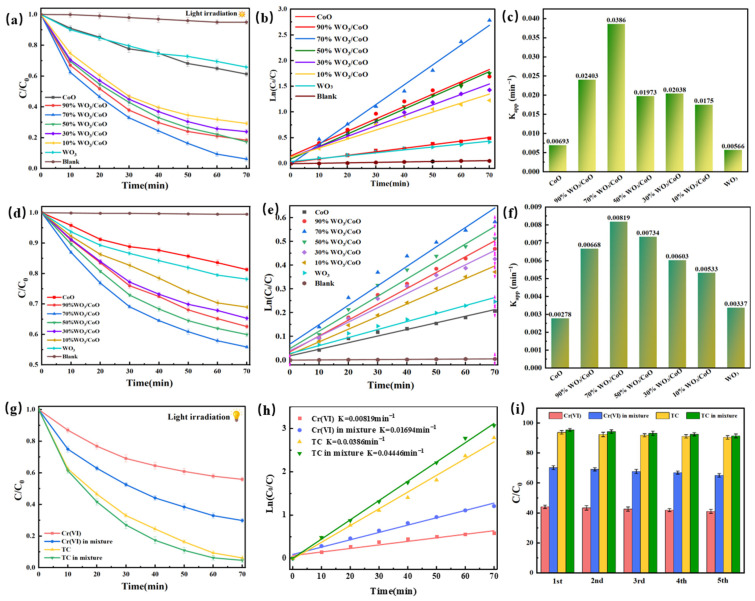
The photocatalytic activity of (**a**) TC, (**d**) Cr(VI), and (**g**) TC and Cr(VI) of the as-prepared samples; first-order reaction kinetics of photocatalytic degradation of (**b**) TC, (**e**) Cr(VI), and (**h**) TC and Cr(VI); the reaction rate constant of photocatalytic activity to (**c**) TC and (**f**) Cr(VI); and (**i**) five-cycle curve of 70% WO_3_/CoO sample for TC and Cr(VI) degradation.

UV-Vis diffuse reflectance spectroscopy was employed to investigate the optical absorption characteristics, band gaps, and energy levels of the CoO, WO_3_, and WO_3_/CoO heterojunctions. In Figure 4a, since pure WO_3_ had a narrow band gap width of 2.9 eV, it had an absorption edge at approximately 466 nm and strong light absorption properties in both the UV and visible spectra [42,45,48]. However, the light absorption intensity of CoO decreased significantly with increasing wavelength, which limited the photocatalytic performance, which was also consistent with the previous studies. The difference was that pure CoO could effectively use UV and visible light [64]. Compared with pure WO_3_, the absorption spectra of the WO_3_/CoO heterojunctions showed a red shift and a significant enhancement of the absorption band edge, indicating that the constructed WO_3_/CoO heterojunctions could effectively enhance the light absorption performance and thus improve the photocatalytic activity [31,41]. As shown in Figure 4b, the forbidden bandwidths of pure WO_3_ and CoO were 2.90 eV and 2.34 eV, respectively, as calculated by the Tauc plot equation (Formula 1). Meanwhile, as shown in Figure 5c,d, the flat-band potentials of pure WO_3_ and CoO were determined using electrochemical Mott–Schottky and displayed at −0.45 V and 0.87 V (vs. NHE), respectively. Due to the negative slope of CoO and positive slope of WO_3_, CoO and WO_3_ were p-type and n-type semiconductors, respectively. Therefore, we can deduce that the potentials of the VB of CoO and the CB of WO_3_ were 0.97 eV and −0.55 eV (vs. NHE), respectively. In summary, the WO_3_ VB potential was 2.35 eV, and the CoO CB potential was −1.37 eV (vs. NHE). In conclusion, it is proven that CoO and WO_3_ could construct Z-scheme WO_3_/CoO p-n heterojunctions in this situation.

(1) Ahν=A(hν-Eg)1/n

α, h, V, Eg, and A are the absorption coefficient, Planck’s constant, incident light frequency, band gap energy, and constant, respectively. The value of N determines the characteristics of transitions in semiconductors, with values equal to 1/2 and 2 representing indirect and direct transitions, respectively.

### 2.5. Photocatalytic Activites

Figure 5 illustrated the photocatalytic performance of pure CoO, pure WO_3_, and 10–90% WO_3_/CoO heterojunctions for the degradation of two monomeric pollutants of TC and Cr(VI) and a mixture of TC and Cr(VI) under the illumination of visible light. Under natural conditions, only 5% of the TC was degraded, as shown in Figure 5a, indicating that it was difficult to achieve the self-degradation of TC in water, while the increase of light time after the photocatalyst injection significantly decreased the concentration of TC. The pure CoO and WO_3_ degraded 38.7% and 34.2% of the TC after 70 min of the photocatalytic reaction. In contrast, 10–90% of the WO_3_/CoO degraded 70.7%, 76.1%, 82.7%, 93.8%, and 81.6% of the TC, respectively. This proved that constructing WO_3_/CoO heterojunctions could effectively improve photocatalytic activity. Among them, 70% WO_3_/CoO heterojunctions exhibited the best performance of the photocatalyst. As shown in Figure 5b, based on the Langmuir–Hinshelwood kinetic model, the kinetic curve had good linear characteristics, indicating that the photocatalytic oxidation of TC follows the quasi-first-order kinetic model. In Figure 5c, it can be seen that, during the degradation of TC, the 70% WO_3_/CoO reaction rate constant is 0.0386 min^−1^, which is 6.26 times and 6.82 times higher than pure CoO (0.00617 min^−1^) and WO_3_ (0.00566 min^−1^), further proving that 70% WO_3_/CoO has the best photocatalytic degradation efficiency for TC. As shown in Figure 5d, the removal of Cr(VI) by pure CoO, WO_3_, and 10–90% WO_3_/CoO after 70 min of reaction was 18.7%, 37.4%, 40.0%, 44.1%, 34.7%, 31.0%, and 21.8%, respectively. Figure 5e shows that the kinetic curve exhibits good linear characteristics, indicating that the photocatalytic removal of Cr (VI) follows a quasi-first-order kinetic model. For other mass ratios of WO_3_/CoO, the reaction rate constant (0.00819 min^−1^) of 70% WO_3_/CoO(Figure 5f) is still the highest, which is 2.95 times and 2.43 times higher than pure CoO (0.00278 min^−1^) and WO_3_ (0.00337 min^−1^), respectively. Considering the coexistence of TC and Cr(VI) in real wastewater, the photocatalytic simultaneous cleanup of TC and Cr(VI) in the mixed solution were measured. Figure 5g demonstrated that the degradation efficiency of pollutants mixed after a 70 min reaction was significantly greater than that of a single TC and Cr(VI). Figure 5h shows that removing TC and Cr (VI) mixed pollutants also follows a quasi-first-order kinetic model. The degradation rate of TC and Cr(VI) increased from 93.8% and 44.13% to 95.35% and 70.2%, respectively, after five cycles, demonstrating that the WO_3_/CoO heterojunction had excellent stability (Figure 5i)_._

### 2.6. Electrochemical Test

**Figure 6:**
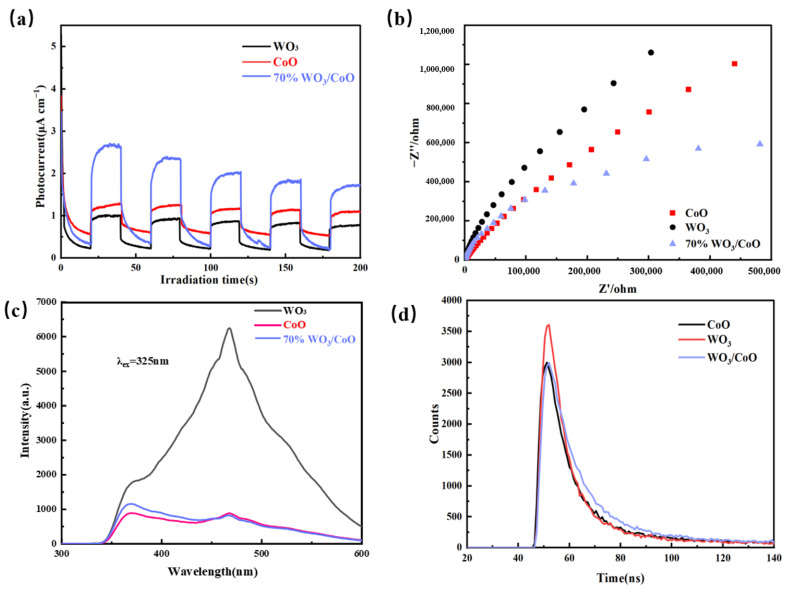
(**a**) Photocurrent behavior, (**b**) electrochemical resistance, (**c**) PL spectrum, and (**d**) time-resolved luminescence decline of CoO, WO_3_, and 70% WO_3_/CoO heterojunctions.

To further investigate and explore the reasons for the improved photocatalytic performance of WO_3_/CoO heterojunctions, the photocurrent- response, electrochemical impedance, photoluminescence spectra, and time-resolved photoluminescence decay curves were used to probe the carrier capture, migration, and electron–hole pair separation efficiency. In Figure 6a,b, the apparent current density of 70% WO_3_/CoO was greater than pure CoO and WO_3_, while the EIS arc radius of 70% WO_3_/CoO arc was the smallest. The above results showed that the construction of the WO_3_/CoO heterojunction can significantly improve the separation efficiency and charge the transfer capability of photogenerated carriers. Subsequently, the 70% WO_3_/CoO heterojunction showed the lowest PL intensity peak in Figure 6c, manifesting that, after successfully constructing the WO_3_/CoO p-n heterostructure, the recombination of electron–hole pairs was effectively suppressed. Moreover, PL lifetimes of photogenerated electron–hole pairs were determined by time-resolved phosphor spectroscopy. The 70% WO_3_/CoO heterojunction revealed a longer lifetime than those of pure CoO and WO_3_ (Figure 6d), which further indicated that the construction of WO_3_/CoO heterojunctions can effectively extend the lifetime of photogenerated carriers and thus restrain the recombination of photogenerated e^−^ and h^+^, which was conducive to improving the photocatalytic performance.

### 2.7. Radical Trapping and ESR

**Figure 7:**
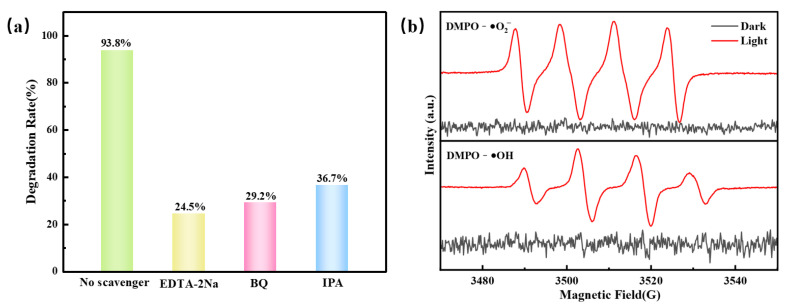
(**a**) Active species trapping experiments for visible photocatalytic degradation of TC by FSCN, and (**b**) ESR spectra of FSCN under visible light and dark conditions.

Active species capture and ESR experiments were performed for determining the significant active species involved in the reaction system and their contribution. EDTA-2Na, BQ, and IPA were selected as scavengers. As shown in Figure 7a, the TC degradation rate reduced from 93.8% to 24.5%, 29.2%, and 36.7%, respectively, indicating that e^−^ and •O_2_^−^ contributed significantly in the reaction, while •OH was also involved in the photocatalytic reaction. To further verify the existence of free radicals, the ESR analysis was performed under visible light. As shown in Figure 7b, no ESR signal regular of DMPO-•O_2_^−^ and DMPO-•OH was observed under dark conditions. However, a series of obvious characteristic peaks was received under light conditions, indicating that •O_2_^−^ and •OH free radicals were involved and played critical roles in photocatalytic reactions.

### 2.8. LC-MS

**Figure 8:**
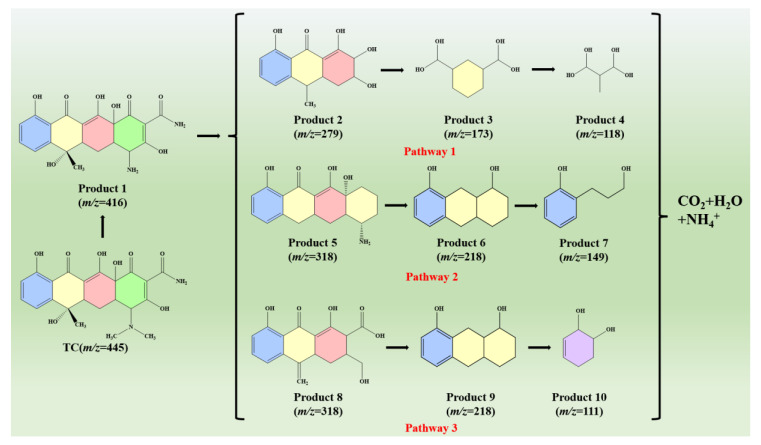
Potential pathways for TC degradation by 70% WO_3_/CoO heterojunctions.

Liquid chromatography–mass spectrometry (LC-MS) was used to validate intermediates to further determine the photocatalytic degradation pathway of TC. The mass spectrum resulting from the degradation of the original TC and corresponding intermediates after 70 min was shown in Figure S3. The protonated tetracycline molecule was represented by the single peak of the original TC sample at *m*/*z* = 445 [65,66,67,68,69]. The degradation of TC into many intermediates was observed after 70 min when the MS peak at *m*/*z* = 445 was significantly reduced, and several new peaks appeared at *m*/*z* = 416, 318, 279, 218, 173, 150, 118, and 111. Based on the combination of the previous studies and the above mass spectra, the structural information of the potential intermediates produced in the photocatalytic process was presented in Table S1. Therefore, Figure 8 shows the potential pathways of TC degradation. Firstly, TC lost two methyl groups to obtain P1 (*m*/*z* = 416). Following this, there are three pathways in further transformation. For Pathway 1, P2 (*m*/*z* = 279) was produced from P1 (*m*/*z* = 416) by ring opening and decarboxylate, and the formation of P3 (*m*/*z* = 173) was formed by ring opening and demethylation from P2 (*m*/*z* = 279), then P4 (*m*/*z* = 118) was achieved through product P3 by ring opening. For Pathway 2, P5 (*m*/*z*= 318) was generated from product P1 (*m*/*z* = 416) by the cleavage of the double bond and the removal of the carboxyl group and amino group. Following this, P6 (*m*/*z* = 218) was converted from P5 (*m*/*z*= 318) via ring opening, deamination, and double-bond oxygen. Subsequently, P7 (*m*/*z* = 149) was obtained by ring opening. For the third pathway, the product P8 (*m*/*z* = 318) was converted from P1 by dehydroxylation, deaminationring, cleavage of bond, and ring opening. Then, the product P9 (*m*/*z* = 218) was obtained by the oxidation of the double bond to the single bond and dehydroxylation. Subsequently, product P10 (*m*/*z* = 111) was converted from product P9 by dehydroxylation, double-bond cleavage, and ring opening. In summary, P4, P7, and P10 were partially mineralized into CO_2_, H_2_O, NH_4_^+^.

### 2.9. Photocatalytic Mechanism

**Figure 9:**
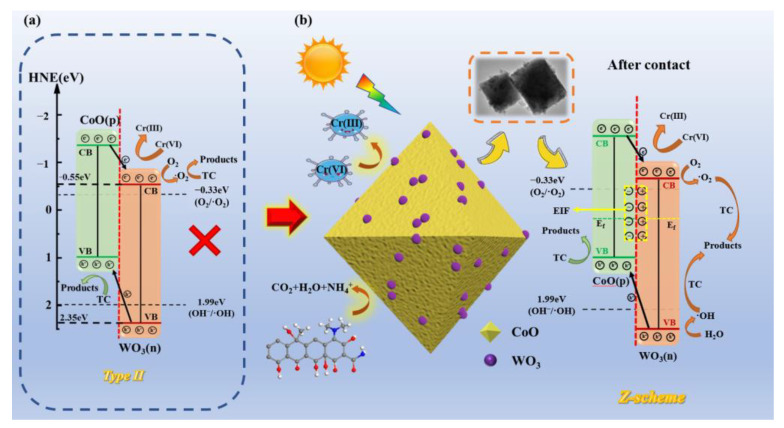
Potential photocatalytic mechanism for TC and Cr(VI) cleanup over WO_3_/CoO p-n heterojunction.

On the basis of the above analysis and results, the degradation mechanism of the TC and Cr(VI) of the Z-scheme WO_3_/CoO p-n heterojunction under visible light was proposed with the following, as shown in Figure 9. In terms of the Mott–Schottky diagram, CB and VB are shown in Figure 9a. It is clear that the band gap positions of WO_3_ and CoO can form type II heterojunctions [70,71]. Electrons transferred to the CB of WO_3_ and CoO, and h^+^ was generated at the VB of WO_3_ and CoO when WO_3_/CoO was irradiated with visible light. Because a p-n heterojunction was formed, photogenerated electrons from the CB of CoO were transferred to the CB of WO_3_ under the influence of the potential difference, while h^+^ from the VB of WO_3_ could be transferred to the VB of CoO. However, the CB potential of WO_3_ (−0.55 eV vs. NHE) is smaller than that of the superoxide radical O_2_/•O_2_^−^(−0.33 eV vs. NHE), and the VB potential of CoO (0.97 eV vs. NHE) is smaller than that of the hydroxyl radical (E(•OH/OH^−^) = +1.99 eV vs. NHE). [6,72,73,74]. This indicated that •O_2_^−^ could be produced during photocatalysis without •OH, which apparently contradicted the ESR results. Therefore, based on the above results and previous literature, a p-n heterojunction that follows the Z-scheme path mechanism was proposed. As shown in Figure 9b, when the p-type CoO made contact with the n-type WO_3_, the electrons on WO_3_ diffused to the CoO surface and suppressed the agglomeration of CoO. At the same time, the Fermi energy of the WO_3_/CoO tended to be balanced due to the electric field created among WO_3_ and CoO. Under visible light irradiation, photogenerated electrons migrated from the CB of WO_3_ to the VB of CoO according to the Z-scheme pathway, preserving the significant redox currents of the CB of CoO and the VB of WO_3_ [75,76,77]. In this case, the main active species produced by the Z-scheme mechanism are •O_2_^−^ and •OH, in agreement with the ESR results. TC decomposes into small molecule substances such as CO_2_, H_2_O, and NH_4_^+^ under the synergistic action of •O_2_^−^, •OH, and h^+^ [5,51,78,79]. Under the act of e^−^ and •O_2_^−^ at the CB of WO_3_, Cr(VI) is reduced to Cr(III). In addition, e^−^ plays a significant role as an electron donor in the photocatalytic reduction of Cr(VI), and the photocatalytic degradation of TC can effectively consume excessive h^+^, thus inhibiting the recombination of the photogenerated e^−^ and h^+^, and ultimately further improving the photocatalytic efficiency of the synergistic removal of Cr (VI) and TC [80,81]. In summary, the Z-scheme WO_3_/CoO p-n heterojunctions could effectively facilitate the separation of photogenerated carriers and improve photocatalytic activity.

## 3. Materials and Methods

### 3.1. Chemicals and Materials

Anhydrous ethanol is purchased from Tianjin Yongda Chemical Reagent Co., Ltd. (Tianjin, China). Sodium tungstate, N-octanol, and cobalt acetate are supplied by Tianjin Damao Chemical Reagent Factory (Tianjin, China), and tetracycline is purchased from Shanghai Baoman Biotechnology Co (Shanghai, China). Nitric acid was purchased from China National Pharmaceutical Group Chemical Reagent Co., Ltd. (Shanghai, China). All experimental water is pure water, and the chemical substances used in the experiment, except for nitric acid, are analytically pure and can be used without further purification.

### 3.2. Characterization

The morphology was evaluated by scanning electron microscopy (SEM) with Phenom ProX, and the microstructure and elements of the composites were analyzed. The transmission electron microscope (TEM) photographs were taken using a transmission electron microscope (FEI, Hillsboro, USA) to observe the structural characteristics of the composites. X-ray diffraction (XRD) of the specimens was performed with Pert-ProMPD/max-γAX-ray (Cu Ka radiation with λ = 1.5406A, 2θ: 10–80°). X-ray photoelectron spectroscopy (XPS) was performed on an Axis ultra-DLD photoelectron spectrometer (Manchester, Britain) to analyze the nature, content, valence, and state of the composite. A Lambda 750 (PerkinElmer, Boston, MA, USA) spectrometer was used to obtain UV-Vis absorption spectra to reflect the optical absorption properties. CoO and WO_3_ were used as a reference to discuss the material’s electronic structure and the semiconductor’s band gap.

### 3.3. Synthesis of WO_3_, CoO, and WO_3_/CoO Heterojunctions

First, 5 mL of 65% concentrated nitric acid (HNO_3_) was slowly added into 25 mL of deionized water, thus obtaining diluted nitric acid. Then, 0.519 g of sodium tungstate (Na_2_WO_4_·2H_2_O) was dissolved in 10 mL of deionized water, and stirred until Na_2_WO_4_·2H_2_O dissolved. Then, as-prepared diluted nitric acid was slowly added into above Na_2_WO_4_·2H_2_O aqueous solution, and ultrasonic-stirred for 30 min. This process changed the sediment from white to faint yellow. The resulting mixture was added to a 50 mL Teflon-lined stainless-steel reactor and heated at a constant temperature of 180 °C for a period of 12 h. At the end of the reaction, the samples were naturally cooled to room temperature, washed alternately with deionized water and ethanol, and placed in a drying oven at 80 °C for 6 h to obtain WO_3_.

**Figure 10:**
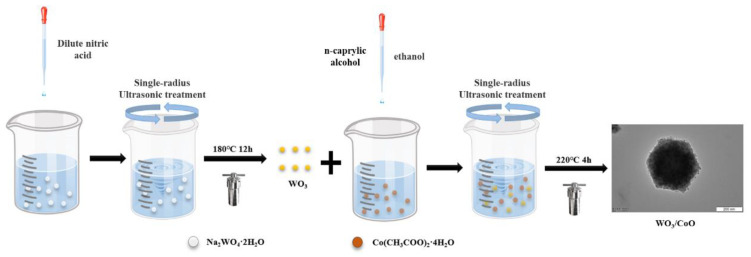
Schematic diagram of synthesis for as-prepared photocatalysts.

The WO_3_/CoO heterojunction were prepared by facile and robust solvothermal synthesis as follows: Take the 90 wt.% WO_3_/CoO heterojunction, for example: 1.84 g cobalt acetate (Co(CH_3_COO)_2_·4H_2_O) was dissolved into the mixture solution of 64 mL octyl alcohol and 16 mL ethanol and stirred for 2 h at room temperature. Then, we added 0.1466 g WO_3_ into the above mixture solution and stirred magnetically for 10 min. Then, the suspension was poured into 100 mL Teflon-lined stainless-steel reactor and heated in oven at 220 °C for 4 h. At the end of the reaction, the samples were naturally cooled to room temperature, obtained by centrifugation, and washed three times alternately with deionized water and ethanol, and finally taken to vacuum-drying oven at 70 °C for 6 h to obtain 90 wt% WO_3_/CoO heterojunctions. Finally, WO_3_/CoO heterojunctions with various mass ratios were produced by adding the corresponding mass ratio of WO_3_ to the above mixed solution under the same conditions and labeled as 10% WO_3_/CoO, 30% WO_3_/CoO, 50% WO_3_/CoO, 70% WO_3_/CoO, and 90% WO_3_/CoO. The fabrication scheme of the WO_3_/CoO p-n heterojunction photocatalyst is presented in Figure 10.

### 3.4. Measurement of Photocatalytic Activity

We separately added 30 mg of 10–90% WO_3_/CoO to the TC solution (50 mL, 40 mg/L) and stirred for 30 min under dark conditions to ensure that the TC reaches adsorption equilibrium on the photocatalyst surface. A group of pure TC solution without any photocatalyst was set as blank control, and its initial concentration was denoted as C_0_. A 300 W xenon lamp with a λ > 420 nm cut filter was used as a visible light source to irradiation solution, with continuous magnetic stirring at atmospheric pressure, and 3 mL of the suspension was taken at 15 min interval and centrifuged to remove the photo catalyst. The experimental temperature was kept at 25 °C to eliminate the effect of temperature on the experiment. The supernatant was measured by setting the maximum wavelength of the UV-Vis spectrophotometer to 357 nm. The degradation rate of the TC solution can be calculated using the following equation:

$$Degradation\left(\%\right)=\left(1-\frac{C}{C_{0}}\right)\times 100\%$$

*C*_0_ is the absorbance of the starting solvent, and *C* is the absorbance of the solvent after the reaction. In order to test the stability of photocatalytic per form, the sample with the best photocatalytic activity were selected for five cycles of experiment; that is, the solution was recovered and centrifuged after the experiment, and cleaned with ethanol and water for several times and dried again to obtain the available photocatalyst, and then the degradation rate of TC was tested using the same method.

Similar to the photocatalytic degradation of the TC experiment, Cr (VI) solution (30 mg/L) was used as the target pollutant. During the irradiation process, 3 mL of the supernatant was taken out every 15 min, and the concentration of Cr (VI) was analyzed at 540 nm using a UV-visible spectrophotometer.

### 3.5. Reactive Radical Trapping Experiments

Similar to the above photocatalysis experiment, keep the original experimental process unchanged. Before the reaction, 1 mmol ethylenediaminetetraacetic acid disodium salt (EDTA-2Na), 1,4-Benzoquinone (BQ), and isopropanol (IPA) captors are added to the TC solution to explore the contribution of superoxide anion radical (O_2_^−^), hydroxyl radical (OH), and hole (h^+^) in the process of photocatalysis experiment.

## 4. Conclusions

In conclusion, Z-scheme WO_3_/CoO p-n heterojunction photocatalysts were successfully prepared by a simple solvothermal method. XRD, SEM, TEM and UV-Vis were used to study the morphology, crystal structure, composition, optical properties, and carrier transfer mechanism. The results show that TC and Cr(VI) can be effectively dislodged by the 70% WO_3_/CoO heterojunction photocatalyst. At the same time, after five cycles, the removal rate of the TC and Cr(VI) of the WO_3_/CoO p-n heterojunction was only slightly reduced, indicating that the heterojunction had excellent stability. Moreover, the 70% WO_3_/CoO heterojunction photocatalyst showed higher photocatalytic activity than pure CoO and WO_3_, and the reaction rate constants of TC degradation are 6.26 times and 6.82 times that of pure CoO and WO_3_, respectively, while the reaction rate constants of Cr(VI) degradation are 2.95 times and 2.43 times that of pure CoO and WO_3_, respectively. Finally, the potential route and photocatalytic process of the degradation of TC and Cr(VI) were determined by an active species trapping experiment, ESR, and LC-ms spectrometry. In summary, new perspectives are provided by these findings in the construction of high-performance photocatalysts for p-n heterojunctions.

## Data Availability

The data of the study can be provided by the corresponding author upon reasonable request.

## Supplementary Materials

The following supporting information can be downloaded at: https://www.mdpi.com/article/10.3390/molecules28124727/s1, Figure S1 SEM images of (a) CoO, (b) WO_3_, and (c) 70% CoO-WO_3_. Survey spectra of 70% WO_3_/CoO heterojunction. Figure S3 Mass spectra of doxycycline hydrochloride of 70% WO_3_/CoO. Figure S4 Nitrogen adsorption desorption isotherm and pore size distribution curve (a–e) 10–90% WO_3_/CoO, (f) CoO, (g) WO_3_; Dark adsorption experiment (h) TC, (i) Cr (VI). Figure S5 (a,b) TEM and HRTEM images of 70% WO_3_/CoO; (c–f) EDX mapping of 70% WO_3/_CoO. Figure S6 (a) TEM images, (b) XRD of 70% WO_3_/CoO after the photocatalytic process. Figure S7 10–90% WO_3_/CoO composite material bandgap. Table S1 Possible intermediate products [82,83,84,85].

## Sample Availability

Samples of the compounds are available from the authors.
