# Supplementary material for: Boosted Tetracycline and Cr(VI) Simultaneous Cleanup over Z-Scheme WO3/CoO p-n Heterojunction with 0D/3D Structure under Visible Light
Source: Molecules. 2023 Jun 13;28(12):4727. doi: 10.3390/molecules28124727 (PMC10304646; doi:10.3390/molecules28124727)
Supplement: Supplementary file 1 [file molecules-28-04727-s001.zip › molecules-2410882-supplementary.pdf]

1. Supplementary Materials:

1.1 Figure

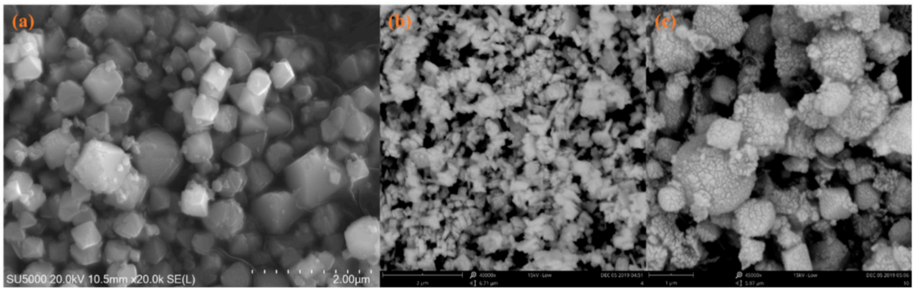

Figure S1 SEM images of (a) CoO, (b) WO_3, and (c) 70% CoO-WO_3.

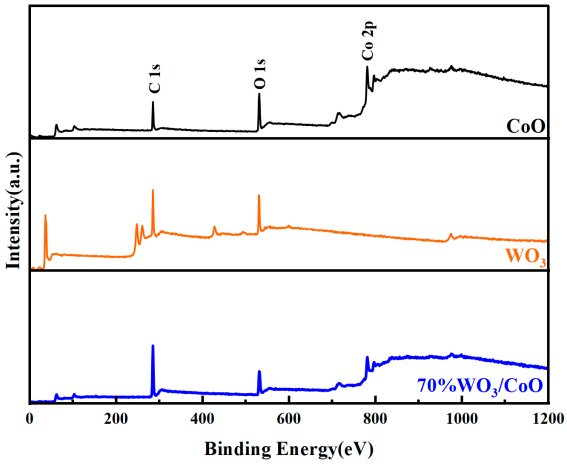

Figure S2 Survey spectra of 70% WO_3/CoO heterojunction

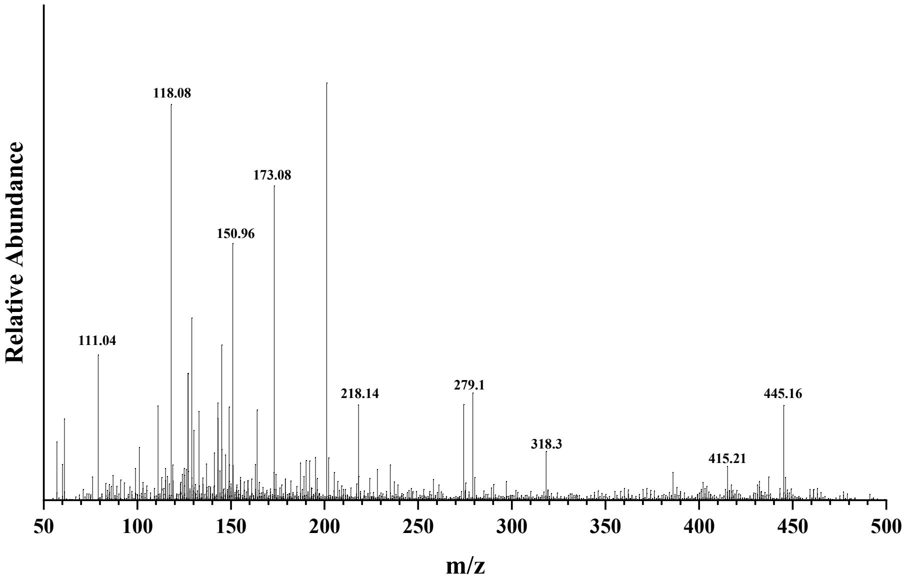

Figure S3. Mass spectra of doxycycline hydrochloride of 70% WO_3/CoO

The N_2 adsorption-desorption isotherm and pore size distribution of WO_3/CoO heterostructure are shown in Figure S4. As shown in the FigureS4(a-g), the specific surface areas of 10-90% WO_3/CoO, CoO, and WO_3 photocatalysts are 13.036045 m^2g^{-1}, 14.685631 m^2g^{-1}, and 15.356614 m^2g^{-1},

10.472091 m^2g^{-1}, 8.672618 m^2g^{-1}, 4.170520m^2g^{-1}, and 8.635148 m^2g^{-1}, respectively. The pore volumes were 0.322932 cm^3g^{-1}, 0.169603 cm^3g^{-1}, 0.149632 cm^3g^{-1}, 0.073650 cm^3g^{-1}, 0.050435 cm^3g^{-1}, 0.034203 cm^3g^{-1}, 0.248725 cm^3g^{-1}, and the pore sizes were 83.125136nm, 41.909145nm, 34.841508 nm, 25.984281 nm, and 22.586335 nm, respectively. This result is closely related to the morphology of the material. WO_3 adheres to octahedral CoO, significantly improving the specific surface area of WO_3/CoO composites. The adsorption properties of the materials are closely related to the specific surface area. As shown in Figure S4(h-i), the adsorption performance of WO_3/coo for TC and Cr (Ⅵ) positively correlates with the specific surface area. It can be seen that the adsorption performance of WO_3/CoO decreased significantly after 30 min, and the adsorption equilibrium was reached. However, the material can only adsorb a few pollutants, and the removal of pollutants mainly depends on the excellent photocatalytic performance of the material, which is also confirmed by the photocatalytic experiment and mechanism.

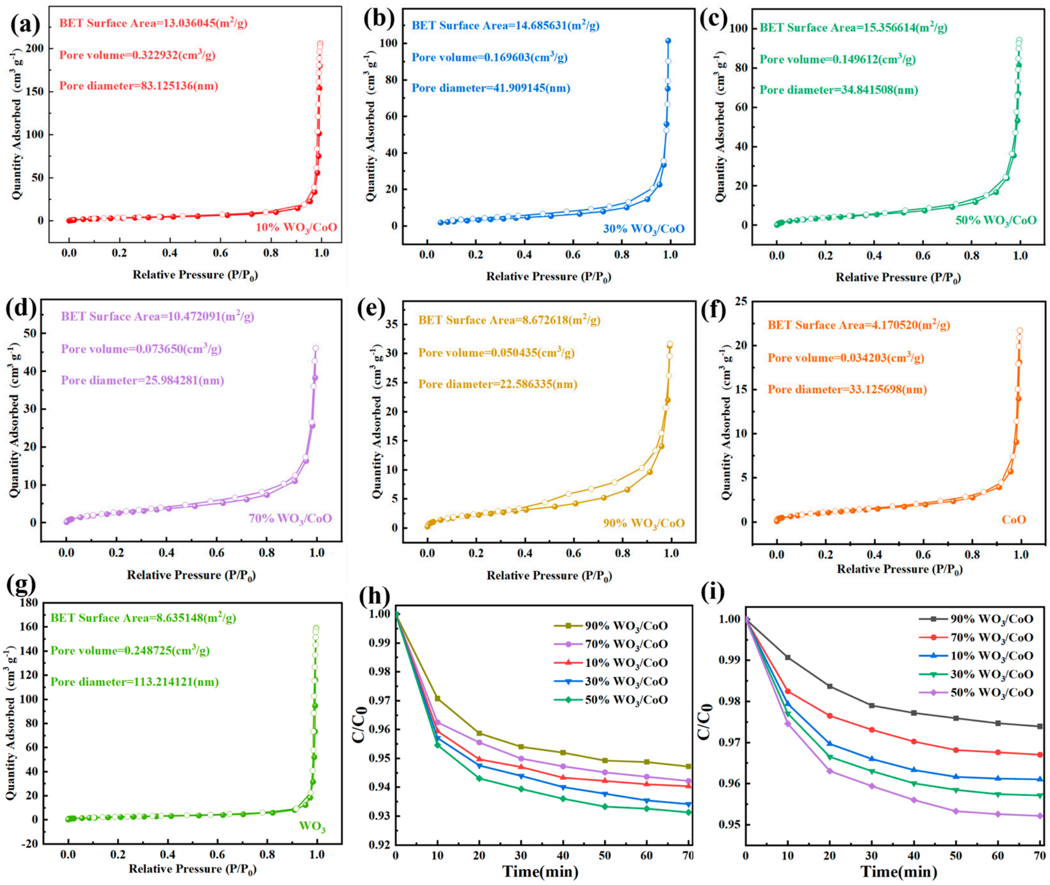

Figure S4 Nitrogen adsorption desorption isotherm and pore size distribution curve (a-e) 10-90% WO_3/CoO, (f) CoO, (g) WO_3; Dark adsorption experiment (h) TC, (I) Cr (VI).

Figure S5(a) shows the TEM image of 70% WO_3/CoO. As shown in the Figure S5(a), granular WO_3 package the surface of octahedral CoO, forming a 70% WO_3/CoO heterojunction, which can provide more photocatalytic reaction sites and further improve photocatalytic activity. Figure S5(b) shows the HRTEM image of 70% WO_3/CoO. The clear stripes indicate that the lattice spacing of nanoparticles is 0.25 nm, which is consistent with the (111) crystal plane of CoO. At the same time, the lattice stripes with a spacing of 0.21 nm can be observed, corresponding to the (202) crystal plane of WO_3. Subsequently, energy dispersive X-ray (EDX) analysis of 70% WO_3/CoO showed (Figure S5 (c-f)) that Co and some O were derived from CoO, while the main element W and some O were derived from WO_3. Co, W, and O are evenly distributed in the 70% WO_3/CoO heterojunction. Therefore, the 70% WO_3/CoO p-n heterojunction was successfully synthesized.

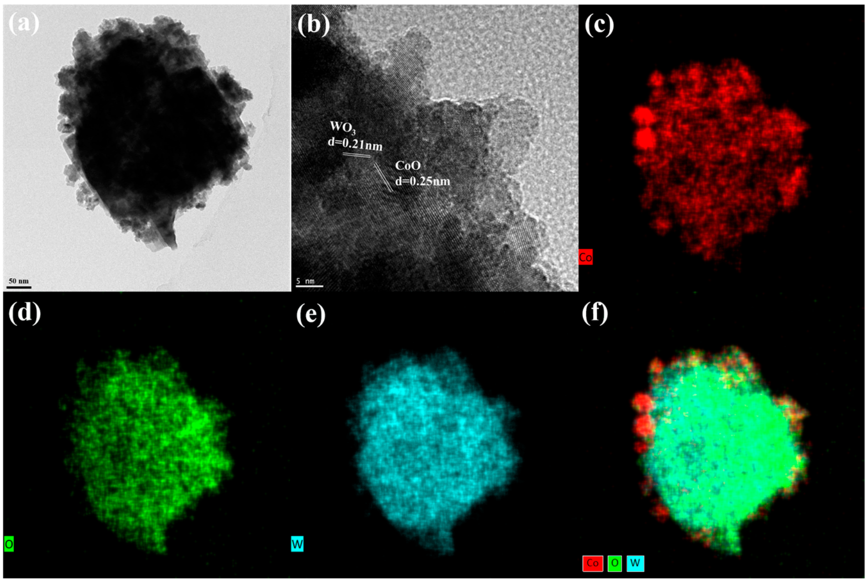

Figure S5 (a-b) TEM and HRTEM images of 70% WO_3/CoO; (c-f) EDX mapping of 70% WO_3/CoO

As shown in Figure S6(a), the TEM image shows that the morphology and structure of 70% WO_3/CoO have not changed significantly after the photocatalytic reaction, and the granular WO_3 is still wrapped on the surface of octahedral CoO, showing excellent structural stability. Through XRD (Figure S6(b)), it can be seen that 70% WO_3/CoO after the reaction still exhibits the characteristics of CoO and WO_3 monomers, and there is no significant change compared to before the reaction, further proving that the morphology and structure of 70% WO_3/CoO have excellent stability.

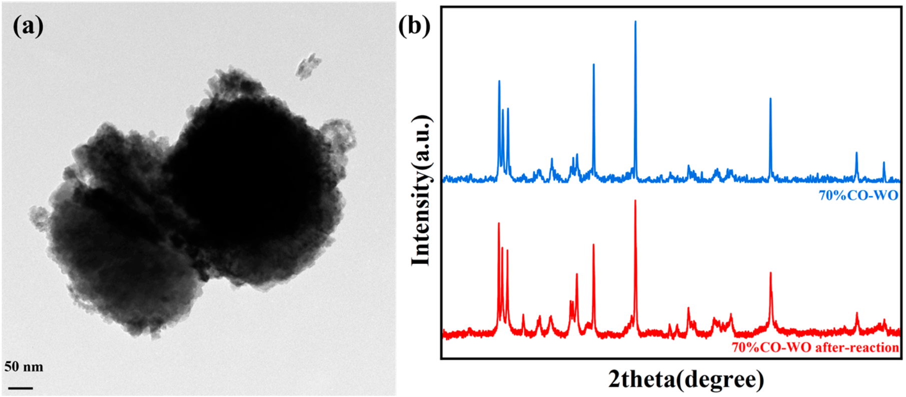

Figure S6 (a) TEM images , (b) XRD of 70% WO_3/CoO after the photocatalytic process

The band gaps of 10-90% WO_3/CoO composite materials were calculated using the Tauc Plot equation, and the band gaps of the composite materials were2.59, 2.33, 2.10, 1.94, and 1.87 eV, respectively.

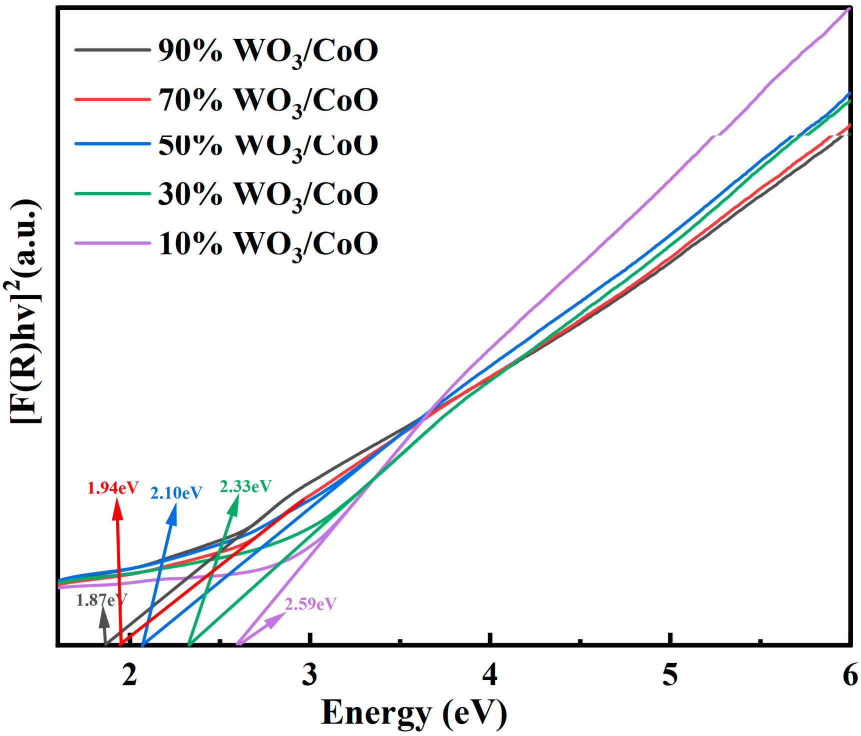

Figure S7 *10-90% WO_3/CoO composite material bandgap1.2 Table*

Table S1 Possible intermediate products

Product	Chemical Formula	Specific Charge(m/z)	Structure
**TC**	$C_{22}H_{24}N_2O_8$	**445**	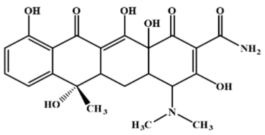
**P1**	$C_{20}H_{20}N_2O_8$	**416**	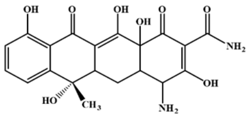
**P2**	$C_{17}H_{18}O_6$	**318**	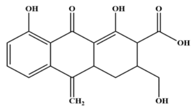
**P3**	$C_{18}H_{21}O_4N$	**318**	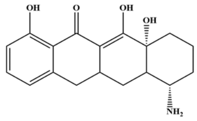
**P4**	$C_{15}H_{16}O_5$	**279**	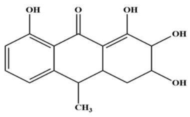
**P5**	$C_{14}H_{20}O_2$	**218**	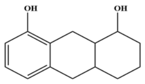

**P6**	$C_8H_{12}O_4$	**173**	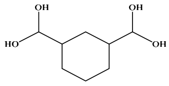
**P7**	$C_9H_{10}O_2$	**149**	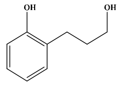
**P8**	$C_6H_8O_2$	**111**	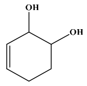
**P9**	$C_4H_6O_4$	**118**	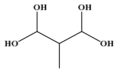

1.3 Material performance comparison

By comparing with other materials, it further proves the performance advantages of 70% WO_3/CoO composite photocatalyst. Shi et al. [1] prepared CoO octahedra decorated with carbon quantum dots for the degradation of tetracycline (TC) in water, with a final removal rate of 87%, significantly lower than 95.35% of 70% WO_3/CoO. The micro-nano spherical CoO/$BiVO_4$ p-n heterojunction photocatalyst prepared by Wang et al. [2] also exhibited excellent photocatalytic performance against TC (87.3%, 90 minutes). Still, it was also lower than 95.35% of 70% WO_3/CoO. The S-scheme WO_3/$CdIn_2S_4$ photocatalyst prepared by Chen et al. [3] can degrade 90% of TC, demonstrating excellent photocatalytic performance but only staying at the level of a single pollutant. Lu et al. [4] conducted research on the removal of mixed pollutants and found that the Z-Scheme type CoO/Bi_2WO_6 p-n heterojunction can achieve synchronous removal of TC and Cr (VI), with removal rates of 90.7% and 57.5%, respectively, still lower than 95.35% and 70.2% of 70% WO3/CoO.

[1] Shi, W.; Guo, F.; Wang, H.; Han, M.; Li, H.; Yuan, S.; Huang, H.; Liu, Y.; Kang, Z. Carbon dots decorated the exposing high-reactive (111) facets CoO octahedrons with enhanced photocatalytic activity and stability for tetracycline degradation under visible light irradiation. Appl. Catal. B Environ.2017, 219, 36–44.

[2] Wang, S.; Zhao, L.; Huang, W.; Zhao, H.; Chen, J.; Cai, Q.; Jiang, X.; Lu, C.; Shi, W. Solvothermal synthesis of CoO/BiVO4 p-n heterojunction with micro-nano spherical structure for enhanced visible light photocatalytic activity towards degradation of tetracycline. Mater. Res. Bull. 2021, 135, 111161.

[3] Pei, C.Y.; Chen, Y.G.; Wang, L.; Chen, W.; Huang, G.B. Step-scheme WO3/CdIn2S4 hybrid system with high visible light activity for tetracycline hydrochloride photodegradation. Appl. Surf. Sci. 2021, 535, 147682.

[4] Lu, C.Y.; Yang, D.Q.; Wang, L.T.; Wen, S.J.; Cao, D.L.; Tu, C.Q.; Gao, L.N.; Li, Y.L.; Zhou, Y.H.; Huang, W. Facile construction of CoO/Bi2WO6 p-n heterojunction with following Z-Scheme pathways for simultaneous elimination of tetracycline and Cr(VI) under visible light irradiation. J. Alloys Compd. 2022, 904, 164046.
